# Experimental manipulation of humidity in a cavity-nesting bird influences ectoparasites' abundance

**DOI:** 10.1017/S0031182022000026

**Published:** 2022-04

**Authors:** F. Castaño-Vázquez, S. Merino, F. Valera, J. Veiga

**Affiliations:** 1 Evolutionary Ecology, Museo Nacional de Ciencias Naturales CSIC, c/José Gutiérrez Abascal no. 2, 28006, Madrid, Spain; 2 Departamento de Ecología Funcional y Evolutiva, Estación Experimental de Zonas Áridas (EEZA-CSIC) Ctra, de Sacramento s/n, La Cañada de San Urbano, 04120, Almería, Spain

**Keywords:** Arid, *Coracias garrulus*, ectoparasites, humidity, temperature

## Abstract

Climate change effects on host–parasite interactions have been poorly studied in arid or semi-arid habitats. Here, we conducted an experiment aimed to increase the temperature inside European roller *Coracias garrulus* nest boxes located in a semi-arid habitat on different nest-site types to look for effects on different ectoparasite abundances and nestling growth. Average nest temperature was slightly higher in heated nests than in control nests, although differences were not statistically significant. However, relative humidity was significantly lower at night in heated nests as compared to control nests. The abundance of sand flies, mites and carnid flies was significantly higher in heated, less humid, nests while biting midge abundance was significantly lower in heated nests. Other ectoparasites were not significantly affected by treatment. Relative humidity was high even in heated nests, reaching more than 60%. Sand fly abundance was higher in nests located in sandstone walls, while mite abundance was higher in isolated farmhouses. In addition, sand fly prevalence was higher in nests located in isolated farmhouses and sandstone walls. Heat treatment, nest-site type or ectoparasite abundances did not affect the nestling body mass, wing length or their growth at different nestling ages.

## Graphical abstract

**Figure:**
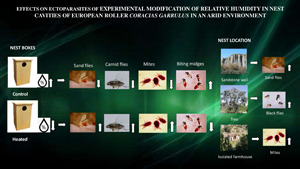

## Introduction

In order to understand how host–parasite interactions can evolve, it is also necessary to know the effects of environmental conditions on these interactions (Poulin, 2007). Temperature can influence the physiology, ecology and the evolution of hosts and parasites (Musgrave *et al*., 2019; Aleuy and Kutz, 2020) while host–parasite interactions can also be altered as a consequence of different factors such as host density (Veiga *et al*., 2020), host breeding seasonality (Merino and Potti, 1995), nest microclimate (Martínez-de la Puente *et al*., 2010), abiotic factors (Martínez-de la Puente *et al*., 2009; Castaño-Vázquez *et al*., 2018) or habitat characteristics (Manzoli *et al*., 2013). Changes in temperature (even small) could therefore alter host resistance or pathogen virulence with important implications on host–parasite interactions (Thomas and Blanford, 2003; Studer *et al*., 2010; Gehman *et al*., 2018). For example, Scharsack *et al*. (2016) showed that a temperature increase can intensify the virulence of tape worms (*Schistocephalus solidus*) by decreasing the resistance to infection of their ectothermic host, the 3-spined stickle back (*Gasterosteus aculeatus*). Gehman *et al*. (2018) found that temperature increase affects positively the reproduction of the parasitic crustacean *Loxothylacus panopaei* reducing infected host survival and producing a net decrease in prevalence on flatback mud crabs (*Eurypanopeus depressus*). Conversely, those ectoparasites that live in tight contact with endotherm hosts could be less affected by environmental conditions (Merino, 2019). With respect to parasites that affect birds, it has been observed that an increase in temperature reduces the relative humidity inside nest boxes, decreasing the population of ectoparasites (Castaño-Vázquez *et al*., 2018, 2021). For instance, Dube *et al*. (2018) found a higher abundance of mites in barn swallow *Hirundo rustica* nests with higher temperature and lower humidity. Yet, Heeb *et al*. (2000) showed that high humidity levels are necessary for the development of ectoparasites such as fleas inside nests. Therefore, humidity levels could play an important role on the prevalence and abundance of ectoparasites inside nests.

Studies on the effects of climate change on host–parasite interactions have been mostly conducted in temperate climatic areas (Merino and Møller, 2010; Møller, 2010) and, consequently, these effects are poorly known in arid or semi-arid areas that are characterized by water scarcity and low rainfall. Arid habitats provide an interesting model to study host–parasite interactions because environmental conditions in these habitats (e.g. lower humidity) could restrict parasite development or their adaptation to these habitats (Veiga and Valera, 2020*b*). Warburton (2020) suggested that arid regions are exemplary places to test new hypotheses on parasite virulence or their transmission and can provide new insights to understand eco-evolutionary relationships between hosts and parasites. On the other hand, drier soils and air in arid habitats could be an impediment for those parasites whose stages require a minimum amount of water for their development or survival. In this respect, other studies have shown that birds in arid habitats have a lower prevalence, abundance and diversity of parasites (Moyer *et al*., 2002; Valera *et al*., 2003). Low precipitation can affect both larval and adult stages of vector populations and therefore the transmission of vector-borne parasitic diseases (Gage *et al*., 2008). In fact, the absence of vectors in different habitats (e.g. marine, saline and arid) is a common explanation for the lack of blood parasite infections there (Bennett, 1992; Figuerola, 1999; Jovani *et al*., 2001; Valera *et al*., 2003). The absence of suitable vectors in arid habitats can be explained by the lack of water that could hinder the completion of their larval phase (Hille *et al*., 2007). Alternatively, Martínez-Abraín *et al*. (2004) suggested that a low host density in arid environments could also explain the absence of blood parasite infections, and Valera *et al*. (2003) proposed that birds inhabiting arid habitats could have a natural resistance to blood parasites. In addition, Tella *et al*. (1999) proposed that bird species with poor immunocompetence may be selected for, or limited to, open habitats in which the prevalence of haematozoa is low (see also Barrientos *et al*., 2014). In the same way, Sehgal (2015) found the evidence of habitat effects on the prevalence and diversity of blood parasites related to variation on factors affecting insect vectors (e.g. temperature changes). Additionally, other studies have shown that the abundance and diversity of haemo- and ectoparasites can be highly variable in arid habitats (Carrillo *et al*., 2007; Belo *et al*., 2012). Probably, low humidity in these habitats could be the limiting factor for ectoparasites (Moyer *et al*., 2002). Another study showed that Mediterranean habitats had a higher prevalence of blowflies *Protocalliphora azurea* in avian nests as a result of higher humidity in comparison to drier habitats (Garrido-Bautista *et al*., 2020). However, Dudaniec *et al*. (2007) found that changes in the prevalence of parasitic fly *Philornis downsi* in Darwin's finches were not due to a direct effect of climate on parasitic fly. In addition, Koop *et al*. (2013) showed that the abundance of parasitic fly *P. downsi* in the nests of the medium ground finch (*Geospiza fortis*) was similar in a dry year as compared to another wet year, indicating that the fly was capable to resist extreme climatic fluctuations. In the same way, Vial *et al*. (2018) showed that soft ticks of the genus *Ornithodoros* can withstand very arid conditions whether the dry seasons were altered with small rains that maintained a minimum of humidity through the year. Therefore, although high temperatures and low humidity in arid habitats are limiting factors for several parasites, some have been able to adapt to these harsh environments and it is thus interesting to study how an increase of temperature or a decrease of humidity will affect the host–parasite interactions in these areas.

Here, we experimentally increase the temperature inside nest boxes occupied by European rollers *Coracias garrulus* in order to assess the effects of such manipulation on the abundance of different ectoparasites attacking bird nestlings and on nestling condition. We also study the potential effects of nest-box location (Eucalyptus trees, sandstone walls and isolated farmhouses) on temperature increase, parasites and hosts.

## Material and methods

### Study area and species

This study was carried out in an area about 50 km^2^ of the Desert of Tabernas (Almería, S. E. Spain, 37°05′N, 2°21′W, 400 m a.s.l.) from 25 April to 27 July 2018. The landscape is mainly made up of badlands, as well as olive and almond groves interspersed among numerous dry stream rivers. The climate is temperate, semiarid Mediterranean with mild winters and hot summers (Lázaro *et al*., 2004). A marked annual variability in temperatures (mean annual temperature around 18°C) and a scarcity of rainfall (mean annual rainfall around 230 mm) are the main characteristics of this study area.

The European roller (*C. garrulus*; hereafter roller) is a hole-nesting Coraciiform that in northern Europe usually nests in natural holes of different trees such as pines and oaks (Cramp and Simmons, 1988). In southern latitudes, rollers nest in cliff cavities and human constructions. However, most of the population in our study area breeds in nest boxes thanks to a nest-box supplementation programme (Václav *et al*., 2011; Valera *et al*., 2019). In our population, incubation takes ~21 days and nestling rollers fledge ~20–25 days (personal observation) after hatching. Egg hatching in rollers is distinctly asynchronous (within-brood nestling ages are in the range 2–10 days; see Václav *et al*., 2008).

During the 2018 breeding season, 59 nest boxes were distributed across the study area on trees, sandstone walls and isolated farmhouses (Valera *et al*., 2019), and were used by several bird species including the roller. These species included the Eurasian collared dove (*Streptopelia decaocto*) and common wood pigeon (*Columba palumbus*) on trees; common kestrels (*Falco tinnunculus*), jackdaws (*Corvus monedula*), rock pigeons (*Columba livia*) and little owls (*Athene noctua*) in sandstone walls; and spotless starlings (*Sturnus unicolor*), house sparrows (*Passer domesticus*), rock pigeons and common kestrels in farmhouses (see Veiga and Valera, 2020*a*). Nest-box dimensions were between 27.5–31 cm height, 25 cm width and 26 cm depth. The entrance hole was of 6 cm in diameter and the thickness of the wall was around 2 cm. Nest boxes on sandstone walls and isolated farmhouses are located in places without vegetation, while nest boxes on trees are covered by dense tree canopy. In addition, it has been shown that nest-box location can contribute to explain the variation in ectoparasite community (e.g. blackflies, biting midges, sand flies and mites) developing inside nest boxes (Veiga and Valera, 2020*a*).

### Heating treatment

Nest boxes were matched according to hatching date, brood size and nest-site location, and within each pair, one was randomly assigned to heated treatment and the other treated as control. Heated nests were supplied with heat mats (70 × 70 mm, 5 V/1.5 W; thermo Flächenheizungs GmbH, Rohrbach, Bavaria, Germany) during 16 days (from day 6 to 21 post-hatching, day 1 = hatching of the first egg). Previous studies have used these heat mats to manipulate the temperature inside nest boxes occupied by blue tits (*Cyanistes caeruleus*) during the nestling period (see Castaño-Vázquez *et al*., 2021). Heat mats were fixed with thumbtacks to the inner wall of the nest and connected to lithium batteries (169.92 × 86.36 × 29.97 mm, 30 A/111 W; Imuto, X6L, China) through a cord with a USB output (48 h autonomy). Batteries were replaced every 2 days to maintain heat mat functioning during the complete experimental period. Control nests were visited with the same frequency than heated nests during the experiment. For each control nest, a cardboard and a string were placed in the nest simulating the heat mat and the cord of heated nests.

A total of 26 nests (13 heated and 13 controls) were included in the experiment. Fourteen nest boxes were located on trees, 8 on sandstone walls and 4 on isolated farmhouses. First, 110 nestlings from 26 nests (55 nestlings from 13 heated nests and 55 nestlings from 13 control nests) were used to explore the effects of treatment on body mass and wing length at 13 days of the older nestling age. After 13 days of age, several nestlings died by unknown reasons in nests from both treatments. In experimental nests, 5 and 3 nestlings died and in control nest 2 and 1 nestlings died at 17 and 21 days of nestling age, respectively. Thus, we used a total of 103 nestlings (50 nestlings from 13 heated nests and 53 nestlings from 13 control nests) and a total of 99 nestlings (47 nestlings from 13 heated nests and 52 nestlings from 13 control nests) to explore the effects of treatment on body mass and wing length at 17 and 21 days of nestling age, respectively.

### Measuring temperature and relative humidity

Temperature and relative humidity inside nest boxes were recorded every hour from day 6 to 21 of nestling age with the aid of data loggers (Hygrochron DS1923; 6 × 17 mm, temperature range: −20 to 85°C; resolution 0.0625°C; humidity range: 0–100% with a resolution of 0.04%; Maxim IC, San Jose, California, USA) that were placed at each nest attached under the nest lid. Once nestlings fledged, sensors were removed and data obtained. One sensor did not register temperature and humidity in one control nest located on a tree. Thus, sample size varied among analyses. Daily average nest temperature and relative humidity from day 6 to 21 of nestling age were calculated and then an average temperature and relative humidity considering average daily values for that period were used for each nest. The same procedure was also used to calculate an average nest temperature and relative humidity for that period at night (from 00:00 to 8:00 h). These hours were selected because it is when temperature decreases to reach minimum values.

### Trapping and quantification of ectoparasites

Biting midges (Fam. Ceratopogonidae), blackflies (Fam. Simuliidae) and sandflies (Fam. Psychodidae) were trapped using sticky traps consisting of a piece of 330 cm^2^ of white vegetal paper smeared with a commercial body oil gel (Johnson's baby oil gel with chamomile; for more details see Tomás *et al*., 2008). Haematophagous mites (Fam. Macronyssidae and Fam. Dermanyssidae) were also trapped using sticky traps but this method is not too effective and was only used as an approximate estimation of the number of mites inside nests. That is, we consider that those traps with more mites correspond to nests where more mites were present. Traps were fixed with thumbtacks under the upper lid of nest boxes during 2 periods of 4 days (see Veiga and Valera, 2020*a*). The first trap was placed from day 13 to 17 post-hatching and then retrieved and substituted by another trap at day 17 that was removed at day 21 post-hatching. Traps were stored in a freezer (−20 °C) and parasites attached were later extracted from the sticky traps using xylol (isomers mixture C_8_H_10_, 99%, density 20/4: 0.862–0.864), identified with the aid of a magnifying glass (NIKON-SMZ645) and counted at the Experimental Station of Arid Zones (Almería, S. E. Spain, 36°50′N, 02°28′W). Once obtained, arthropods were preserved in ethanol absolute (100%) until quantification. Parasite abundance in each nest was estimated as the sum of parasites captured in both sticky traps.

In addition, the number of ectoparasitic flies (*Carnus hemapterus*) was counted directly on the body surface of each nestling twice and both counts were averaged (see Veiga and Valera, 2020*a*; Veiga *et al*., 2020). *Carnus hemapterus* is a nidicolous ectoparasite that parasitizes nestlings during its imago stage. The imago remains on the nestlings and in the nest debris, so that sticky traps are not suitable to estimate the abundance of carnid flies. This method of visual estimation has been found to be reliable (Roulin, 1998). The number of carnid flies that remained in the nest debris was also counted and added to the sum of the number of carnid flies in all nestlings to get the total number of *Carnus* flies inside the nest (see Veiga *et al*., 2020). This estimation was done when the older nestling was 13 days old because *Carnus* infestation is higher at this stage (see Václav *et al*., 2008; Václav and Valera, 2018).

### Nestling body condition

Nestlings were measured and weighed at 13, 17 and 21 days post-hatching and banded with numbered aluminium rings at 17 or 21 days post-hatching. Body mass of nestling was measured with an electronic balance (±0.1 g) and wing length with a ruler (±0.1 mm). Average body mass and wing length of nestlings for each nest were calculated at 13, 17 and 21 days of nestling age.

### Statistical analyses

To test for the effect of temperature manipulation on nest-box microclimate, a 2-way analysis of variance (ANOVA) test was performed with average temperature and relative humidity for the day and night (see above) as dependent variables and nest-site type, treatment (heat and control) and their interaction as independent variables. Temperature and relative humidity were checked to comply with normality assumptions. Relative humidity was transformed as the arcsine of the square root to attain normality.

Generalized linear models with a negative binomial distribution and log link function were used to compare ectoparasite abundances between nests assigned to different treatments or from nest boxes located in different nest-site types. Counts of each ectoparasite group (i.e. blackflies, sandflies, carnid flies, biting midges and mites) were used as dependent variables, and nest-site type and treatment were introduced as independent variables. Then, we used a likelihood-ratio *χ*^2^ test to compare the current model *vs* the null (intercept) model. The likelihood-ratio *χ*^2^ test assesses the overall significance of the model, indicating whether the explained variance in our data was significantly higher than the unexplained variance. A significant result of this test indicates that the current model fits the data better than the null model (Sokal and Rohlf, 1998) and we explored the significance of the independent variables and their interactions. Conversely, a non-significant result suggests that the model is not sufficient to determine model fit for the predictors and, therefore, the null model is better than the model with the predictors. Contingency tables (3 × 2) and *χ*^2^ or Fisher exact tests were used to analyse differences in the prevalence of each ectoparasite group (i.e. blackflies, sandflies and carnid flies) and nest-site types. The 2 × 2 contingency tables and *χ*^2^ tests were used for analyses of differences between treatment and prevalence of ectoparasites.

To test the relationships between ectoparasites and average nestling growth variables, we used analyses of covariance (ANCOVAs) with the differences in average body mass or wing length between days of measurements as dependent variables and abundance of each ectoparasite group and brood size as covariates while controlling by treatment and nest-site type as independent variables. That is, heat treatment and parasite effects on nestling mass and wing length were evaluated at each date of measurement (13, 17 and 21 days post-hatching) and also for growth differences between nestling ages (13–17; 13–21 and 17–21 days post-hatching). We also tested for differences in nestling mortality between treatments. In all tests, we conducted a backward stepwise procedure to reduce the model to the significant variables. Graphics and statistical analyses were performed with STATISTICA 7 (StatSoft Inc., 2005) and IBM SPSS Statistics for Windows (IBM Corporation, 2019).

## Results

### Effects of heat treatment on temperature and relative humidity in roller nests

Average nest temperature did not show significant differences with treatment, nest-site type or their interaction (ANOVA, *P* > 0.1 for all cases). Average nest relative humidity also did not differ between treatments, nest-site type and their interaction (ANOVA, *P* > 0.1, for all cases; Table 1). Since we did not find any significant differences in temperature and relative humidity between heated and control nest boxes throughout the day (24 h), we assessed the treatment effect only at night (from 00:00 to 8:00 h), when temperature is not so influenced by sunlight and is decreasing and reaching the lowest daily values into the nest boxes. Thus, all data reported hereafter refer to temperature and relative humidity conditions at night, unless otherwise stated.

**Table 1.** Differences in average temperature (°C) and relative humidity (%) during the period 6–21 days between heated and control nest boxes of rollers *Coracias garrulus* during the whole day (24 h) and during the night (from 0:00 to 8:00 h) respectively

	Day		Night	
	Control (*n* = 13) Mean ± s.d.	Heated (*n* = 13) Mean ± s.d.	Control (*n* = 13) Mean ± s.d.	Heated (*n* = 13) Mean ± s.d.
Temperature	25.89 ± 2.01	26.51 ± 1.29	21.51 ± 1.15	22.13 ± 1.37
Relative humidity	54.77 ± 12.85	48.85 ± 4.85	67.63 ± 11.66	60.12 ± 5.42

Average night nest temperature did not show significant differences between heated and control nests (ANOVA, *F*_1,24_ = 1.57, *P* = 0.222). However, nest relative humidity was significantly lower in heated nests as compared to control nests (ANOVA, *F*_1,22_ = 5.54, *P* = 0.027; Table 1) and significantly higher in trees than in sandstone walls or isolated farmhouses (ANOVA, *F*_2,22_ = 4.01, *P* = 0.032). The interaction term was not significant and was eliminated from the analysis (ANOVA, *P* > 0.4).

### Effects of treatment on ectoparasite abundance

**Fig. 1.**
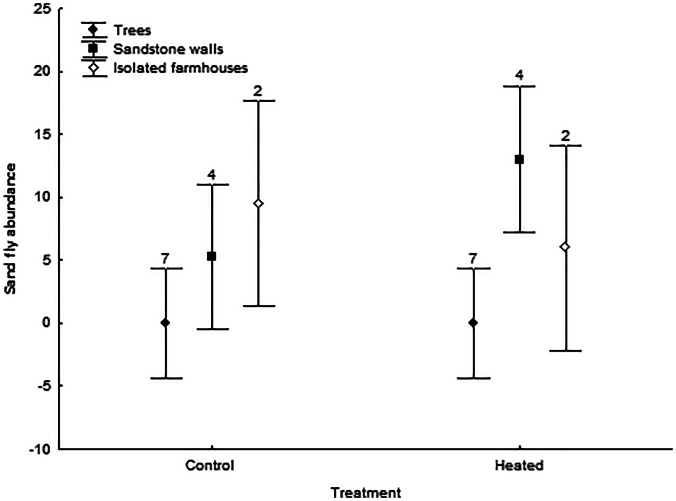
Differences in the abundance of sand flies in roller nests by treatment and nest-site type. Means ± intervals of confidence at 95% are shown. Sample size (number of nests) is shown over the bars.

All models were significant (*P* < 0.02 in all cases) and therefore we explored the significance of explicative variables for each parasite. Sand fly abundance was significantly higher in heated nests as compared to control nests (B = 0.46, *F*_1,20_ = 86.00, *P* < 0.001, Table 2). In addition, sand fly abundance was significantly higher in nests from sandstone walls than in nests from trees or from isolated farmhouses (B = −32.02, *F*_1,20_ = 111.70, *P* < 0.001, Table 3). Furthermore, the interaction between nest-site type and treatment was significant, although this effect was only due to a higher abundance of sand flies in heated nests from sandstone walls compared to control nests (B = −0.45, *F*_1,20_ = 89.89, *P* < 0.001, Fig. 1).

**Table 2.** Prevalence (Prev.) and mean abundance (MA) and standard deviation of ectoparasites in control and heated nest boxes of rollers *Coracias garrulus*

	Control (*n* = 13)		Heated (*n* = 13)	
	Prev. (%)	MA ± s.d.	Prev. (%)	MA ± s.d.
Sand flies	38.46	3.07 ± 5.67	46.15	4.92 ± 8.29
Mites	7.69	0.09 ± 0.30	15.38	10.15 ± 36.01
Blackflies	76.92	9.31 ± 15.89	76.92	24.85 ± 53.58
Biting midges	23.07	0.46 ± 0.97	15.38	0.31 ± 0.85
Carnid flies	92.30	30.42 ± 40.80	100	51.13 ± 36.40

**Table 3.** Prevalence (Prev.) and mean abundance (MA) and standard deviation of ectoparasites in nest boxes of rollers *Coracias garrulus* located on different nest-site types

	Trees (*n* = 14)		Sandstone walls (*n* = 8)		Isolated farmhouses (*n* = 4)	
	Prev. (%)	MA ± s.d.	Prev. (%)	MA ± s.d.	Prev. (%)	MA ± s.d.
Sand flies	0.00	0.00 ± 0.00	87.50	9.12 ± 10.09	100	7.75 ± 3.20
Mites	14.30	0.21 ± 0.57	0.00	0.00 ± 0.00	25.00	32.5 ± 65.00
Blackflies	92.90	28.92 ± 51.54	62.50	2.62 ± 3.58	50.00	4.5 ± 7.14
Biting midges	35.70	0.71 ± 1.13	0.00	0.00 ± 0.00	0.00	0.00 ± 0.00
Carnid flies	100	26.50 ± 28.04	100	67.06 ± 45.04	75.00	38.20 ± 44.13

Blackfly abundance did not show significant differences between heated and control nests (B = −1.60, *F*_1,20_ = 1.04, *P* = 0.319, Table 2). However, blackfly abundance was significantly higher in nests from trees than in nests from sandstone walls and isolated farmhouses (B = 1.74, *F*_2,20_ = 6.02, *P* = 0.009, Table 3). In addition, the interaction between nest-site type and treatment was not significant (B = 0.56, *F*_2,20_ = 0.79, *P* = 0.466).

Biting midge abundance was significantly lower in heated nests as compared to control nests (B = 0.01, *F*_1,20_ = 14.37, *P* = 0.001, Table 2), although these differences disappeared when nest-site type was removed from the analysis (B = 0.40, *F*_1,24_ = 0.18, *P* = 0.673). In addition, the interaction between nest-site type and treatment was not analysed because biting midge abundance in sandstone walls and farmhouses was zero.

Carnid fly abundance in nests was significantly higher in heated nests as compared to control nests (B = −3.07, *F*_1,10_ = 9.67, *P* = 0.011, Table 2), although these differences disappeared when the non-significant effect of nest-site type (B = −0.59, *F*_1,14_ = 0.74, *P* = 0.402) was removed from the analysis. The interaction between nest-site type and treatment was significant, although this effect was only due to a higher abundance of carnid flies in heated nests from isolated farmhouses (B = −0.59, *F*_2,10_ = 1.29, *P* = 0.037).

Mite abundance was significantly higher in heated nests as compared to control nests (B = −3.93, *F*_1,22_ = 10.60, *P* = 0.004, Table 2). In addition, mite abundance was significantly higher in nests from isolated farmhouses than in nests from trees or from sandstone walls (B = −4.51, *F*_1,22_ = 9.95, *P* = 0.005, Table 3). The interaction between nest-site type and treatment was not analysed because mite abundance in sandstone walls was zero.

In addition, the prevalence of each ectoparasite group did not show significant differences between heated and control nests (*χ*^2^ test: *P* > 0.05, in all cases, Table 2). Similarly, the prevalence of blackflies was similar between nest-site types (*χ*^2^ test; *P* > 0.05 in all cases, Table 3). However, sandstone walls and isolated farmhouses had a higher prevalence of sand flies than trees (*χ*^2^ = 22.41, *P* < 0.001, Table 3). Although not statistically significant, the prevalence of carnid flies tended to be higher in sandstone walls and trees than in isolated farmhouses (*χ*^2^ = 5.72, *P* = 0.057, Table 3). The prevalence of biting midges and mites did not show significant differences between nest-site types (Fisher's exact test: *P* > 0.05 for both cases).

### Effects of heat treatment and ectoparasites on body condition of nestlings

Neither average nestling body mass nor wing length was significantly related to heat treatment or nest-site type at 13, 17 and 21 days of nestling age (ANCOVA, *P* > 0.05 for all cases). Similarly, the differences in average nestling body mass or wing length between different nestling ages (13–17; 13–21 and 17–21 days post-hatching) were not significantly related to heat treatment, nest-site or ectoparasite abundances (ANCOVA, *P* > 0.05 for all cases). Nestling mortality did not show significant differences between heated and control nests at 17 and 21 days of nestling age (Fisher's exact test: *P* > 0.05 for both cases).

## Discussion

Most works have assessed the effects of climate change on host–parasite interactions in temperate areas (Merino and Møller, 2010; Møller, 2010), whereas few studies have assessed such effects in warmer areas (Veiga and Valera, 2020*b*). In this study, we experimentally manipulated temperature in roller *C. garrulus* nests during the nestling period to investigate the effect of an increase of temperature on ectoparasite abundance in nests. We failed to experimentally create significant differences in nest temperatures between nests probably due to high fluctuations due to outer ambient temperatures. However, the treatment reduced significantly the relative humidity inside the nest during night hours. It is surprising that relative humidity was so high (more than 60%) in roller nests but this fact could help nestling rollers to survive in a dry environment while developing inside a cavity. In fact, Maziarz (2019) suggested that birds could modify humidity levels inside nest cavities as a strategy to avoid the risk of overheating inside nests. In addition, an elevated nest relative humidity could explain the absence of an effect of heat treatment on temperature.

On the other hand, average nest temperature was similar among different nest-box locations. However, relative humidity was significantly higher in nests from trees than in nests from isolated farmhouses or sandstone walls. Probably, nest relative humidity was higher on trees because their dense canopy covered nest boxes (see Veiga and Valera, 2020*a*) and direct solar radiation on nest boxes could have been less effective. In any case, the effects of treatment on ectoparasite abundances or nestling condition in our study were mainly attributed to significant differences in nest relative humidity.

Ectoparasite abundance in roller nests was significantly higher in heated nests compared to control nests except in the case of biting midges, which were significantly less abundant in heated nests compared to control nests, and blackflies, which did not vary significantly between treatments. However, carnid fly and biting midge abundance in roller nests were not affected by heat treatment when the effect of nest-site type was removed from the analysis. In the same way, Veiga and Valera (2020*a*) showed that nest location could explain the variation of ectoparasite community in roller nests. Previous studies have shown that ectoparasite abundance in avian nests varied when nests were subjected to temperature increase and the consequent humidity decrease (Dawson *et al*., 2005; Castaño-Vázquez *et al*., 2021). For example, Prudhomme *et al*. (2015) found a higher abundance of sandflies *Phlebotomus ariasi* in the south of France when temperature reached 35°C and relative humidity decreased. In this study, the authors also observed that *P. ariasi* activity finds its optimal nocturnal temperature ranges between 20 and 25°C, just in the range found in nests in our study at night. In the same way, Branco *et al*. (2013) found a higher sand fly density in central Portugal associated to higher temperature (25.6°C) and low relative humidity (60% *vs* the usual 70–80%). Similarly, other studies have suggested that rainfall or high relative humidity can negatively affect the sand fly activity in Mediterranean regions (Gálvez *et al*., 2010; Dantas-Torres *et al*., 2014; Prudhomme *et al*., 2015).

On the other hand, the abundance and prevalence of sand fly in roller nests were significantly higher in sandstone walls and isolated farmhouses than in trees. Furthermore, the interaction between nest-site type and treatment in relation to sand fly abundance clearly indicates that heat treatment had an effect on sandstone walls. More sandflies were collected in heated than in control nests and more in heated nests on sandstone walls than in control nests on trees. Adult sand flies often inhabit rock crevices, caves and animal burrows or human dwellings (Alexander, 2000; Lawyer and Perkins, 2000). In our study area, sand flies are able to colonize these microhabitats that appear mostly in sandstone walls and isolated farmhouses. In agreement with our results, Veiga and Valera (2020*a*) also found a higher abundance of sandflies in roller nests located on sandstone walls and isolated farmhouses. The preference of sand flies for these nest types could be due to their lower humidity as compared to the ones on trees but also to an effect of the treatment reducing humidity in heated nests on sandstone walls. Similarly, the higher abundance of mites in heated nests compared to control nests could be due to the fact that these arthropods prefer higher temperature and lower humidity for development. Although humid environments could offer optimal conditions for mite growth (Chen and Mullens, 2008), other studies have shown that a higher temperature and lower humidity could positively affect the mite population in avian nests (Dube *et al*., 2018). In fact, haematophagous mites find its optimal development and can complete the egg-to egg cycle in just 7 days under conditions of high temperature (28–30°C) and medium humidity. In addition, red mites *Dermanyssus gallinae* can live up to 8 months away from their hosts and can withstand drier conditions, but do not tolerate high humidity (Chauve, 1998).

In our study area, we found a higher abundance of mites in nests on isolated farmhouses compared to nests on trees or on sandstone walls. In fact, relative humidity was significantly lower in boxes on isolated farmhouses and sandstone walls compared to the ones on trees. Probably, a higher abundance of mites in nest boxes on isolated houses could also be due to their use (previous to the arrival of rollers) by other bird species such as spotless starlings or house sparrows which could have transported these arthropods to nests (Veiga and Valera, 2020*a*). In addition, we found a higher abundance of blackflies in nest boxes on trees compared to the ones on sandstone walls or isolated farmhouses. Similarly, Veiga and Valera (2020*a*) found that blackflies had a higher preference for roller nests located on trees. In addition, Černý *et al*. (2011) showed that blackflies could select tree canopies for resting. Veiga and Valera (2020*a*) reported differences in the prevalence of ectoparasites between nest-site types. However, we only found a higher prevalence of sand flies in nest boxes on sandstone walls and isolated farmhouses compared with the ones on trees using the nests sampled in this study.

Nestling body mass and wing length growth between different nestling ages did not vary between treatments or nest sites. Similarly, Castaño-Vázquez *et al*. (2021) did not find significant differences in blue tit nestling body condition (e.g. mass and wing length) when nests were subjected to temperature increase at 2 different latitudes. However, Vaugoyeau *et al*. (2017) found an increase of body mass in great tit *Parus major* nestlings subjected to temperature increase in a population from the north of France. In addition, other studies found that an increase in temperature in avian nests negatively affected the nestling body mass (Rodríguez and Barba, 2016; Andreasson *et al*., 2018). Although differences in relative humidity and the number of ectoparasites between heated and control nests were significant (see Tables 1 and 2), it was not enough to detect significant differences in nestling body condition. Heated nests had less humidity and more ectoparasites (e.g. sand flies, mites and carnid flies) compared to control nests. This was a surprising effect because reduction in humidity is detrimental for ectoparasites, at least in less arid environments (see e.g. Castaño-Vázquez *et al*., 2018, 2021). Alternatively, Moyer *et al*. (2002) proposed that low humidity could have little effect on blood-feeding individuals due to the high water content on their diet. However, humidity levels in roller nest are very high and apparently detrimental or unattractive for ectoparasites. An increase in parental effort in the nest affected by more parasites may compensate for the effect of these on nestlings (Bouslama *et al*., 2002; Merino, 2010).

Despite the higher abundance of ectoparasites such as sand flies and mites in heated nests, average nestling body mass and wing length were not affected. Previous studies did not find a relation between brood mass and the abundance of ectoparasites in roller nests (see Veiga and Valera, 2020*a*; Veiga *et al*., 2020). It is possible that parasite load in roller nests was not high enough to show changes on average nestling growth. For example, Merino and Potti (1995) found that nestling body mass was lower in pied flycatcher *Ficedula hypoleuca* nests with a higher abundance of mites. In the same way, Weddle (2000) found that haematophagous mites *Pellonyssus reedi* abundance in house sparrow nests affected negatively the nestling body mass. Alternatively, it is possible that the deleterious effects of ectoparasites would have been compensated by the feeding effort of parents (Møller, 1993; Merino *et al*., 1998). In that case, ectoparasite effects do not appear to significantly affect the body mass of nestlings or their wing length.

On the other hand, given that we did not find a positive relationship between heat treatment and average nestling growth, it is possible that nestlings in worse body condition were not affected by ectoparasites in nests. For example, several studies have found that the ectoparasitic fly *C. hemapterus* preferred roller nestlings in a better body condition (Václav *et al*., 2008; Václav and Valera, 2018). In the same way, different studies have shown that ectoparasite abundance in nests was determined by host body size (e.g. Marshall, 1981; Valera *et al*., 2004) because larger hosts could provide higher resources to ectoparasites.

Based on these results, we can conclude that a slight increase of temperature reduced relative humidity at night inside nest cavities of rollers and affected positively and significantly some ectoparasites such as sand flies and mites inside nests. Nest-box location could be an essential factor to predict ectoparasite abundance inside nests. In addition, average nestling body mass or wing length was not affected by heat treatment, nest-site or ectoparasite abundances. Thus, our results suggest that climatic conditions in arid environments could serve to understand the adaptations of multitude of parasites to these areas and highlight the importance of high humidity level for some parasites in roller nests.

## Data Availability

The datasets generated for this study are available on request to the corresponding author.
